# 2015 ESC guidelines for the management of acute coronary syndromes in patients presenting without persistent ST-segment elevation: comments from the Dutch ACS working group

**DOI:** 10.1007/s12471-016-0939-y

**Published:** 2016-12-13

**Authors:** P. Damman, A. W. van ’t Hof, J. M. ten Berg, J. W. Jukema, Y. Appelman, A. H. Liem, R. J. de Winter

**Affiliations:** 1 Academic Medical Center, University of Amsterdam, Amsterdam, The Netherlands; 2 Isala Klinieken, Zwolle, The Netherlands; 3 St Antonius Hospital Nieuwegein, Nieuwegein, The Netherlands; 4 Leiden University Medical Center, Leiden, The Netherlands; 5 VU University Medical Center, Amsterdam, The Netherlands; 6 St Franciscus Gasthuis Rotterdam, Rotterdam, The Netherlands

**Keywords:** NSTE-ACS guidelines, NVVC ACS working group statement

## Abstract

On behalf of the Dutch ACS working group, we discuss multiple recommendations which have been implemented in the 2015 ESC guidelines for the management of acute coronary syndromes (ACS) in patients presenting without persistent ST-segment elevation.

## Introduction

The 2015 ESC guidelines for the management of acute coronary syndromes (ACS) in patients presenting without persistent ST-segment elevation were presented at the European Society of Cardiology (ESC) Conference 2015 in London and published in the European Heart Journal [1]. Compared with the 2011 version, multiple recommendations have been implemented which we discuss from a Dutch perspective.

## High-sensitive troponin

**Fig. 1:**
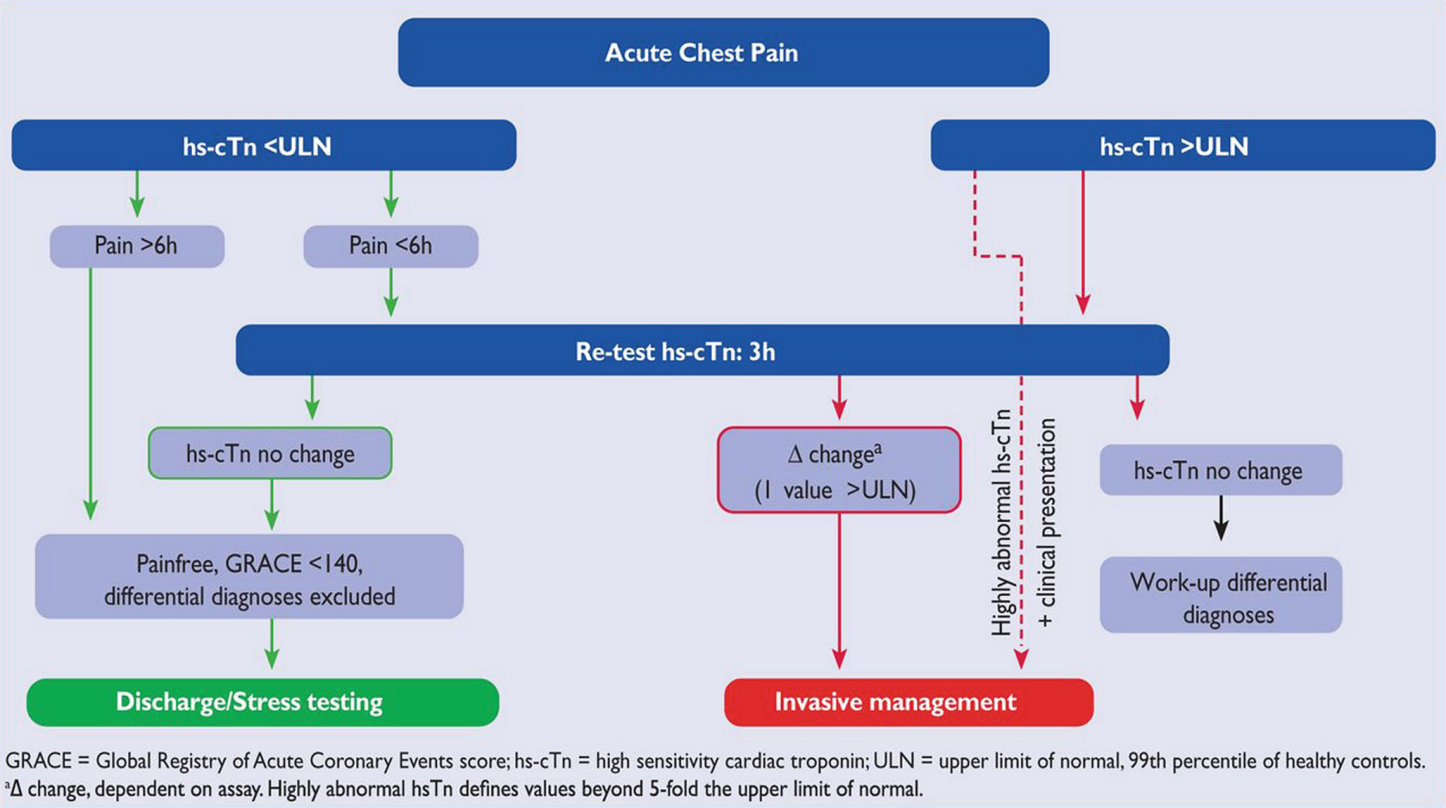
0 h/3 h rule-out algorithms using high-sensitivity cardiac troponin assays in patients presenting to the emergency department with suspected non-ST-elevation myocardial infarction (With permission of Oxford University Press (UK)© European Society of Cardiology, www.escardio.org)

**Fig. 2:**
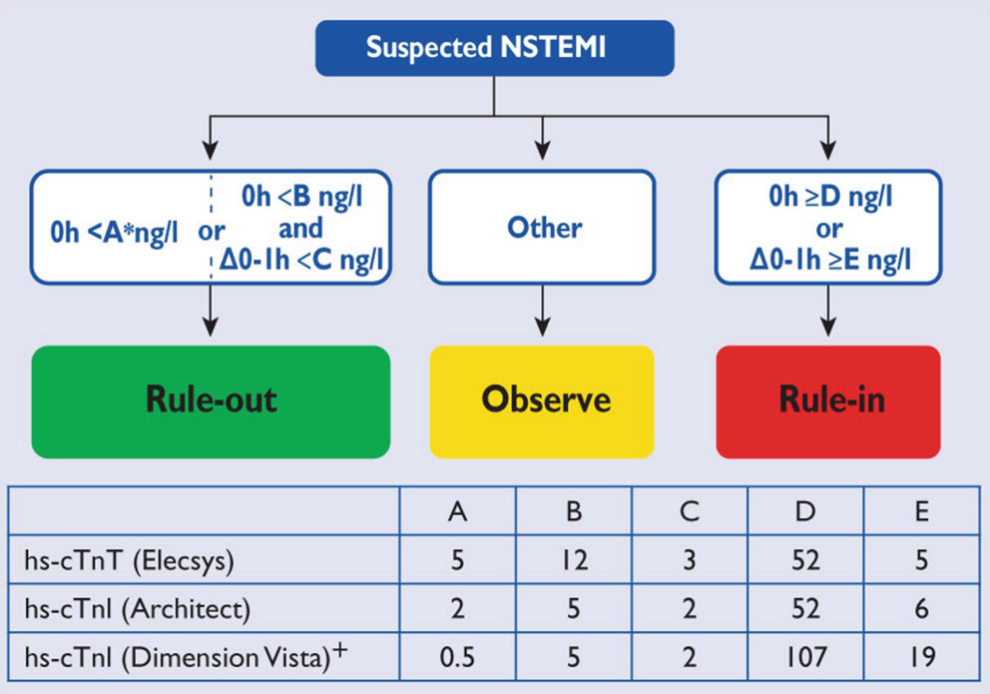
0 h/1 h rule-in and rule-out algorithms using high-sensitivity cardiac troponin assays in patients presenting to the emergency department with suspected non-ST-elevation myocardial infarction (With permission of Oxford University Press (UK)© European Society of Cardiology, www.escardio.org)

The introduction of high-sensitive cardiac troponin has led to a better detection and quantification of myocardial injury. Both the absolute value and change in troponin over time provide information on cardiomyocyte injury, and several studies have assessed the sensitivity and specificity of these measurements [2]. In the 2015 guidelines, algorithms are presented for rule-in and rule-out of non-ST-elevation myocardial infarction (NSTEMI) with the use of high-sensitive cardiac troponin (Figs. 1 and 2). We advise to use these high-sensitive troponin assays and incorporate the aforementioned algorithms in daily practice in the Netherlands.

## Platelet aggregation inhibition at admission

When NSTE-ACS is diagnosed, there is an indication for treatment with dual platelet aggregation inhibitors (acetylsalicylic acid and a P2Y12 inhibitor). For patients managed conservatively, the 2015 guidelines advise to use ticagrelor over clopidogrel. While the 2011 ESC guidelines recommended starting dual antiplatelet therapy (DAPT) as soon as possible before coronary angiography [3], the most recent guidelines are less strict suggesting to initiate the P2Y12 inhibitor either before or after coronary angiography. This change is based on the results of the ACCOAST study, in which patients with NSTE-ACS, who were scheduled to undergo catheterisation, were randomised to pretreatment with prasugrel or placebo [4]. Pretreatment with prasugrel did not reduce the rate of major ischaemic events up to 30 days but increased the rate of major bleeding complications. Until more evidence is available, the current guidelines thus provide the opportunity to individualise treatment and postpone the initiation of P2Y12 inhibition in patients with known coronary anatomy or electrocardiographic changes suggesting three-vessel disease or left-main disease and therefore a suspected indication for early coronary artery bypass surgery (CABG). In patients with a low to intermediate bleeding risk and a high probability of subsequent percutaneous coronary intervention (PCI), pretreatment with clopidogrel or ticagrelor might be useful.

## Triple antithrombotic therapy

**Fig. 3:**
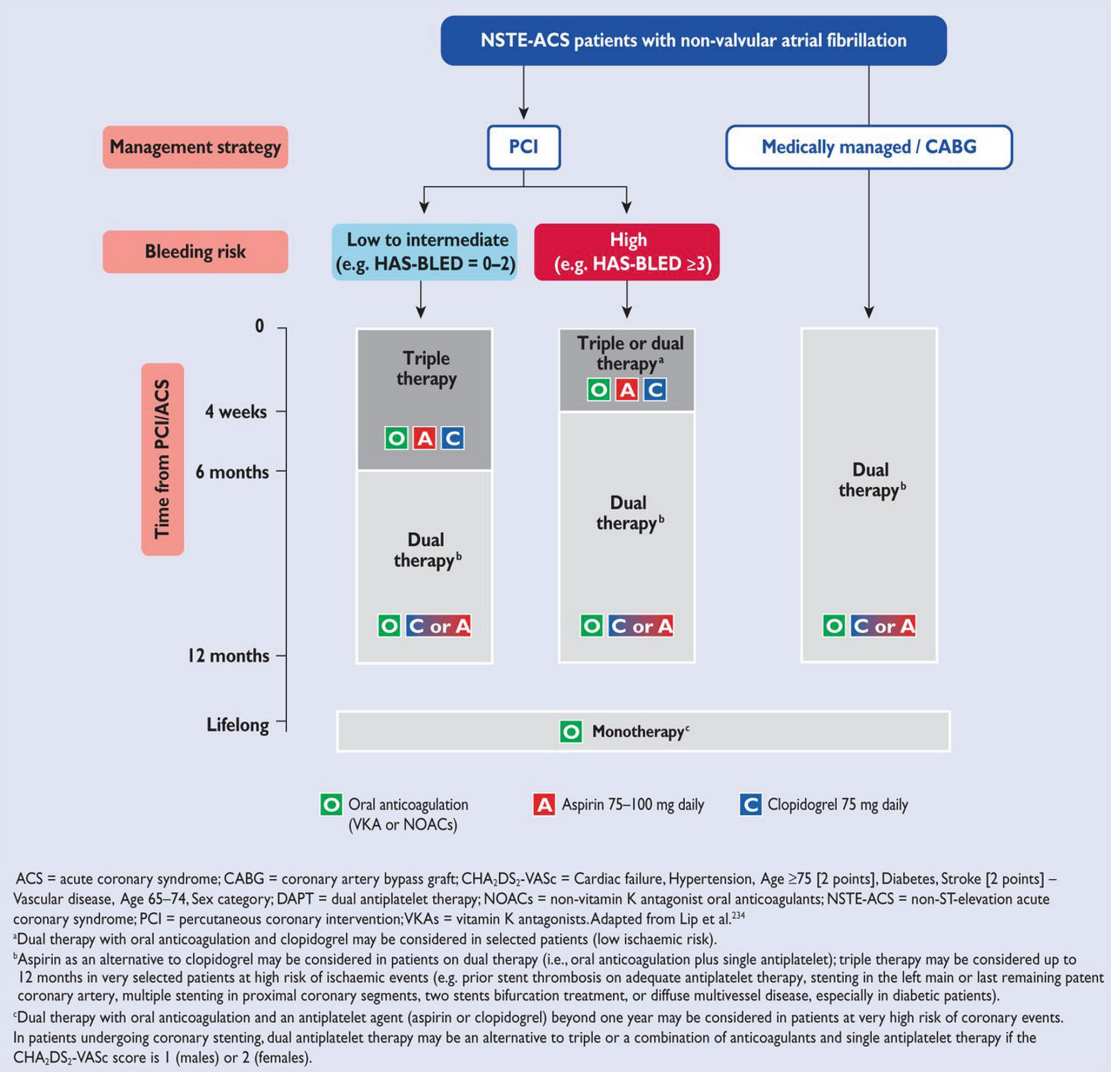
Antithrombotic strategies in patients with non-ST-elevation acute coronary syndromes and non-valvular atrial fibrillation (With permission of Oxford University Press (UK)© European Society of Cardiology, www.escardio.org)

A subset of patients with NSTE-ACS have indications for long-term (non-vitamin K) oral anticoagulation ([N]OAC) such as atrial fibrillation or mechanical heart valves. In combination with ACS, regardless of the performance of PCI, there is an indication for triple therapy (DAPT with [N]OAC). Long-term triple therapy is, however, associated with increased bleeding outcomes [5], and a subsequent increased mortality. Therefore, individualised treatment is necessary in which the ischaemic risk is weighed against the bleeding risk. The current ESC guidelines provide a useful approach in which both the ischaemic and the bleeding risk are taken into account (Fig. 3). In medically managed patients or patients undergoing CABG, a combination of single antiplatelet aggregation therapy and (N)OAC is recommended. If the NSTE-ACS patient undergoes PCI, one or six months of triple therapy is recommended depending on the bleeding risk. After one or six months, a combination of single antiplatelet aggregation therapy and (N)OAC is continued. The Dutch WOEST trial has demonstrated that dual therapy after PCI might be adequate for the prevention of ischaemic events, with a reduction of bleeding events [6]. Combinations of (N)OAC with the stronger platelet aggregation drugs prasugrel or ticagrelor is discouraged because of the excessive bleeding risk. We advise to follow the treatment algorithm as shown in Fig. 3, and emphasise to individualise the treatment based on the ischaemic and bleeding risk. Furthermore, there is room for improvement with regards to the communication between the interventional cardiologist performing PCI and the treating physician, especially regarding ischaemic and bleeding risk. Complex coronary interventions, such as multiple stent constructions and the placement of bioabsorbable vascular scaffolds, might require more intensive and longer treatment with DAPT also when combined with (N)OAC. Otherwise, monotherapy with (N)OAC is recommended after 1 year.

**Fig. 4:**
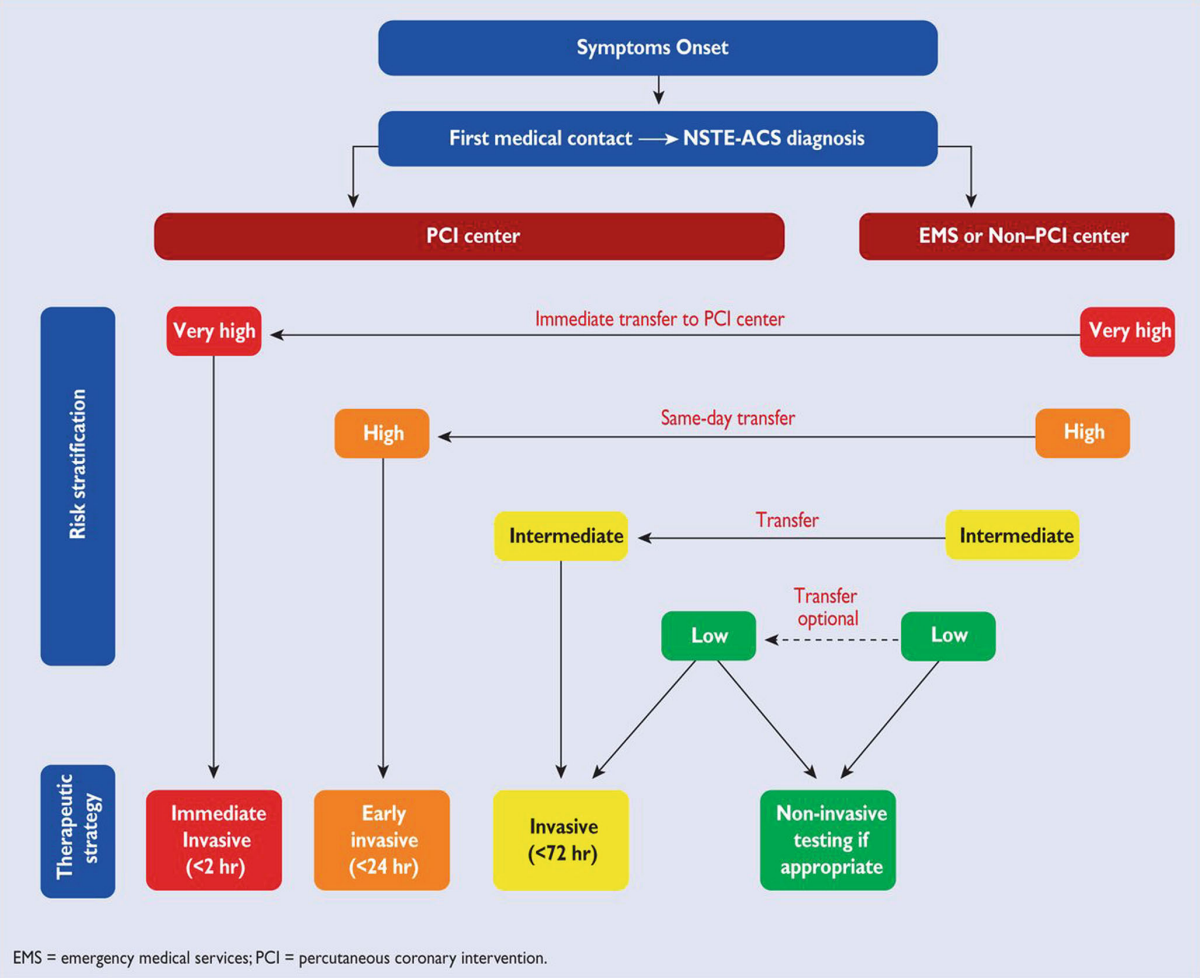
Selection of non-ST-elevation acute coronary syndrome treatment strategy and timing according to initial risk stratification (With permission of Oxford University Press (UK)© European Society of Cardiology, www.escardio.org)

## Same-day transfer in high-risk patients

Comparable with the 2011 ESC guidelines, the current guidelines mention that the decision for and timing of invasive coronary angiography is based on risk stratification and the assessment of the risks related to the procedure (Fig. 4).

### Very high-risk patients

Patients at very high risk, including haemodynamic instability or cardiogenic shock, recurrent or ongoing chest pain refractory to medical treatment, life-threatening arrhythmias, mechanical complications of MI, acute heart failure, or recurrent dynamic ECG changes, should be referred for urgent PCI. Urgent PCI is defined as within 2 h of admission, analogous to primary PCI in ST-segment elevation MI.

### High-risk patients

It is recommended that high-risk patients are transferred from a non-PCI centre to a PCI centre for coronary angiography within 24 h. High-risk patients are defined as patients with a rise and fall in cardiac troponin comparable with MI, dynamic ST- or T‑wave changes, or a GRACE score >140.

The ACS working group does not consider referral within 24 h to be a necessity for the Dutch situation, based on the following considerations. First, the scientific basis for the recommendation is weak as it is only based on two meta-analyses of randomised trials and a retrospective analysis of the ACUITY trial [7, 8]. Both meta-analyses showed no benefit for the hard endpoints mortality, nonfatal MI or major bleeding, but only a reduction in refractory ischaemia. Although the TIMACS trial demonstrated a beneficial effect of early intervention in a high-risk subgroup (GRACE >140), this was only a hypothesis-generating result in a trial which did not show a significant reduction of the primary endpoint death or myocardial infarction [9]. Second, the Dutch situation is markedly different from that in many other European countries since the majority of Dutch cardiology departments are equipped with a catheterisation laboratory where diagnostic coronary angiography is routinely performed in ACS patients. After diagnostic angiography, patients are discussed in a heart team and only those patients suitable for PCI and CABG are referred to an interventional centre. We do not know whether referring all NSTEMI-ACS patients for undergoing catheterisation leads to over-treatment by performing ad-hoc PCI. Third, the current experience of non-PCI centres in the Netherlands as well as the results of the ICTUS trial show us that a more conservative (selective invasive) treatment of NSTE-ACS patients is also a good option [10]. Fourth, same-day transfer of patients based on a rise and fall in cardiac troponin might result in unnecessarily transferring patients with other pathology such as myocarditis or a type II MI (demand ischaemia) associated with heart failure of arrhythmias.

Other issues for implementing the 2015 ESC guidelines are that the Dutch hospitals and ambulance services do not have sufficient capacity for same-day transfer and that not performing the diagnostic angiogram in non-PCI centres could endanger the viability of the catheterisation laboratory in these hospitals. Subsequently, this might have important consequences for the role of the acute cardiac care and coronary care units and supply of patients. The ACS working group considers reducing catheterisation capacity in non-PCI centres not applicable if this is not based on proven-health benefits.

In this regard, from 2017, the ACS working group will inventorise and evaluate the current NSTE-ACS treatment in the Netherlands in collaboration with the Netherlands Society of Cardiology (NVVC), general practitioners, ambulance service, the NVVC ACS Connect project and the NCDR (national cardiovascular data registry). First results are expected in 2018.
